# Supplementary material for: Enhanced Figure of Merit in Bismuth-Antimony Fine-Grained Alloys at Cryogenic Temperatures
Source: Sci Rep. 2019 Oct 17;9:14892. doi: 10.1038/s41598-019-50325-7 (PMC6797736; doi:10.1038/s41598-019-50325-7)
Supplement: Supplementary file 1 — Supplementary Information [file 41598_2019_50325_MOESM1_ESM.pdf]

Supplementary materials for

Enhanced Figure of Merit in Bismuth-Antimony Fine-Grained Alloys at Cryogenic Temperatures

Sheng Gao[1, a)], John Gaskins[2], Xixiao Hu[1], Kathleen Tomko[2], Patrick Hopkins[2, b)], S. Joseph Poon[1, c)]

[1] Department of Physics, University of Virginia, Charlottesville, VA 22904-4714

[2] Department of Mechanical and Aerospace Engineering, University of Virginia, Charlottesville, VA 22904-4259

Contents:

S1. Electron backscatter diffraction (EBSD) images for $Bi_{85}Sb_{15}$ alloy

S2. Simulation method and parameters for $Bi_{85}Sb_{15}$ alloy

S3. Decoupling of electron and hole contributions to thermoelectric transport

S4. Magnetic fields simulation and correction of TE measurements with magnet plates

a) sg5jk@virginia.edu

b) peh4v@virginia.edu

c) sjp9x@virginia.edu

S1. Electron backscatter diffraction (EBSD) images for $Bi_{85}Sb_{15}$ alloy

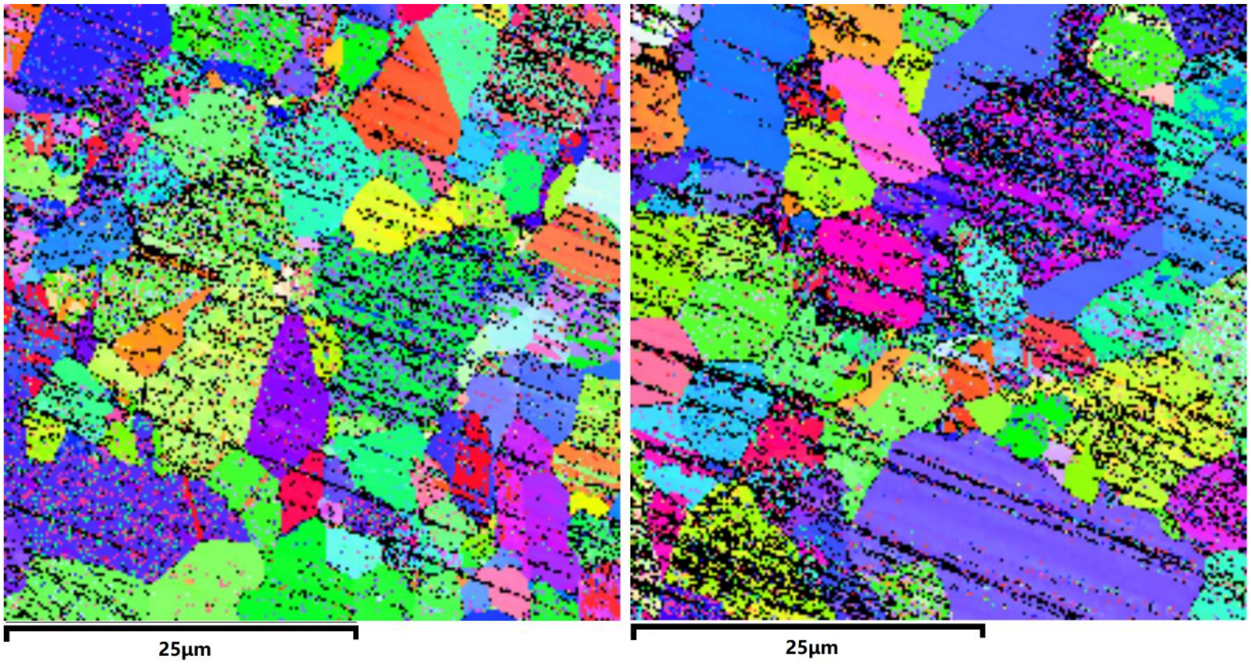

Fig. S1 Electron backscatter diffraction (EBSD) images of SPS Bi85Sb15 sample. fine grains of micron size can be seen. Black lines are scratches from polishing.

S2. Simulation method and parameters for $Bi_{85}Sb_{15}$ alloy

In order to calculate the carrier concentrations (electron concentration *n* and hole concentration *p*), the charge neutrality equation was applied:

$$p + N_d^+ = n + N_a^- \tag{1}$$

where N_d^+, N_a^- are the ionized donor and acceptor concentration, respectively.

For $Bi_{85}Sb_{15}$, the Hall coefficient data at 50 K showed $|n - p| = |N_d^+ - N_a^-| \approx 1.6 * 10^{23}\ m^{-3}$. It was based on the assumption that the donors and acceptors were fully ionized above 50 K, and N_d^+ and N_a^- would stay constant as the temperature changed.

The equations for the carrier concentrations were given by:

$$n = \int_{0(CBM)}^{\infty} f \cdot D_n(E) dE \tag{2}$$

$$p = \int_{-\infty}^{0(VBM)} f \cdot D_p(E) dE \tag{3}$$

where f is the Fermi-Dirac distribution function, $D_{n\,(p)}(E)$ is the density of states of the conduction (valence) band, CBM and VBM are the conduction band minimum and valence band maximum, respectively.

The Matthiessen's rule was used to calculate the scattering rate $\frac{1}{\tau_{total}}$ in the undoped $Bi_{85}Sb_{15}$.

$$\frac{1}{\tau_{total}} = \frac{1}{\tau_{acoustic}} + \frac{1}{\tau_{impurity}} \tag{4}$$

The first term $\frac{1}{\tau_{acoustic}}$ represents the acoustic deformation potential scattering (ADP), which is related to the electron-lattice interaction. The trend of the mobility caused by the ADP scattering is proportional to $T^{-1.5}$. The equation for $\frac{1}{\tau_{acoustic}}$ was given by[1]:

$$\frac{1}{\tau_{acoustic}} = \frac{\pi \varepsilon_{ac}^2 k_B T}{\hbar c_l} g(E) \tag{5}$$

where ε_{ac} is the acoustic deformation potential, c_l is the longitudinal elastic constant, $g(E) = \frac{1}{2\pi^2}\left(\frac{2m_b^*}{\hbar^2}\right)^{\frac{3}{2}}\sqrt{E}$ is the density of states effective mass of a single valley, $m_b^* = \frac{m_d^*}{N_v^{2/3}}$ and N_v is the number of valleys in the Fermi surfaces. The values for those parameters are listed in Table S1.

Parameters	Electrons	Holes
m_d^* (m_e)	0.203	0.483
m_c^* (m_e)	0.025	0.084
N_v	3	6
c_l (N/m^2)	6.6×10^{10}	6.6×10^{10}

Table S1. The parameters in the simulation of undoped $Bi_{85}Sb_{15}$. The acoustic deformation potential ε_{ac} is around 20 eV.

The second term $\frac{1}{\tau_{impurity}}$ was coming from the ionized impurity scattering in this n-type material. It has the form $\tau_{impurity} = \tau_0(E/k_BT)^{3/2}$, where τ_0 is constant.[1] The mobility trend will be proportional to $T^{1.5}$ if the ionized impurity scattering dominates, resulting in an increase in resistivity at low temperatures.

The following equation is usually used to describe the relationship between resistivity and energy gap in a semiconductor:

$$\rho = \rho_0 \exp\left(E_g/2k_BT\right) \quad (6)$$

where ρ_0 is a constant and E_g is the band gap.

In view of the impurity scattering term, instead of directly using Eq. 6 to determine the band gap at low temperature (<100K), we have used a bandgap value of 14 meV based on the result from single crystal Bi-Sb alloy.[2] The plots of the carrier concentration and mobility are shown in Fig. S2. It was found that the rising of the resistivity of our SPSed $Bi_{85}Sb_{15}$ was not entirely caused by the carrier concentration related to the band gap; it was also due to the ionized impurity scattering which significantly changed the mobility trend below 100K.

S3. Decoupling of electron and hole contributions to thermoelectric transport

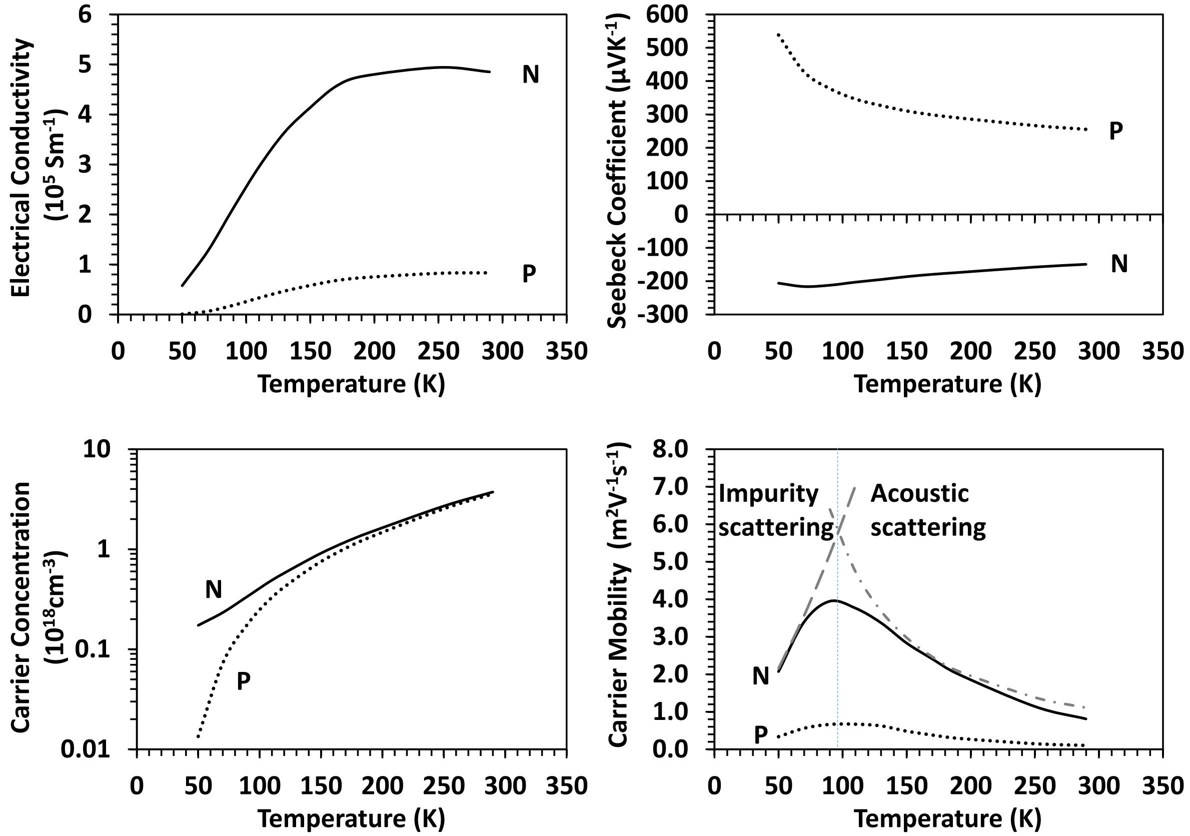

Fig. S2 Simulation results of decoupled n, p channel transport properties for SPSed $Bi_{85}Sb_{15}$: Electrical conductivity, Seebeck coefficient, carrier concentration and carrier mobility. Ionized impurity scattering trend and acoustic deformation potential scattering trend in different temperature region were also shown in the carrier mobility plot.

Based on the electronic structure of bismuth, the quasi-ellipsoid centered at the L-point of Brillouin zone where the electrons are located are strongly elongated along a direction tilted by an angle ϕ_e out of the binary-bisectrix plane (ϕ_e=6 ± 0.2° at 4.2 K)[3]. This highly anisotropic shape led to unusually small effective masses along with two directions that resulted in a very high electron mobility[4], especially when compared with the hole mobility due to the much larger effective hole mass. Therefore, the undoped $Bi_{85}Sb_{15}$ system behaved as a strong n-type semiconductor dominated by electrons. By analyzing the transport properties using a two-band effective mass model, we were able to quantitatively decouple the contributions from electron and hole channels. Results are shown in Fig. S2. The electrical conductivity and Seebeck coefficient components can be calculated using Eq. 7 and Eq. 8. σ_n and S_n are the electrical conductivity component and Seebeck coefficient component of the electron channel, and σ_p and S_p are the electrical conductivity component and Seebeck coefficient component of the hole

channel. m_c^* is the conductivity effective mass, the values can be found in Table S1. μ is the chemical potential and E_g is the band gap. The same scattering mechanisms were used for electrons and holes to calculate the TE transport properties.

$$\sigma = \sigma_n + \sigma_p = -\frac{2e^2}{3m_{c,n}^*}\int_0^\infty \frac{\partial f_n}{\partial E} D_n(E) E\tau_n dE - \frac{2e^2}{3m_{c,p}^*}\int_0^\infty \frac{\partial f_p}{\partial E} D_p(E) E\tau_p dE \quad (7)$$

$$S = \frac{S_n\sigma_n + S_p\sigma_p}{\sigma_n + \sigma_p} = \frac{\frac{2e}{3Tm_{c,n}^*}\int_0^\infty \frac{\partial f_n}{\partial E} D_n(E)E(E-\mu)\tau_n dE - \frac{2e}{3Tm_{c,p}^*}\int_0^\infty \frac{\partial f_p}{\partial E} D_p(E)E\left(E+E_g+\mu\right)\tau_p dE}{-\frac{2e^2}{3m_{c,n}^*}\int_0^\infty \frac{\partial f_n}{\partial E} D_n(E)E\tau_n dE - \frac{2e^2}{3m_{c,p}^*}\int_0^\infty \frac{\partial f_p}{\partial E} D_p(E)E\tau_p dE} \quad (8)$$

At low temperature, the alloy is extrinsic with electrons dominating the mobility, and ionized impurity scattering (~$T^{1.5}$) is the main scattering mechanism. While above 110K, acoustic deformation potential scattering (~$T^{-1.5}$) becomes the primary scattering mechanism, as shown in Fig. S2. There was a turning point for S_n at 70 K. This could be approximately explained using Mott's equation[5] for a single band:

$$S \sim m_d^* T\left(\frac{\pi}{3n}\right)^{2/3} \quad (9)$$

where m_d^* is the density of state effective mass, *n* is the carrier concentration.

At low temperature, the system is n-type extrinsic, the number of electrons does not change dramatically with temperature. So, $|S_n|$ increases nearly linearly as the temperature increased. However, at higher temperatures, intrinsic excitation must be considered. The bipolar effect becomes increasingly important and the magnitude of the total Seebeck coefficient $|S_{total}| = \left|\frac{\sigma_n S_n+\sigma_p S_p}{\sigma_n+\sigma_p}\right|$ decreases. It is worthy to note that S_p was found to have a relatively larger value compared with $|S_n|$ due to two main factors for hole carriers: (1) larger density of states effective mass than electrons; (2) lower carrier concentrations than electrons.

S4. Magnetic fields simulation and correction of TE measurements with magnet plates

The magnetic field generated by the magnet plates was simulated using standard textbook formulae. The two uniform magnet plates were divided into infinitely small magnetic dipoles $\mathrm{d}\vec{\mathrm{m}}$ with magnetization $\vec{\mathrm{M}}$ along the x-direction, the coordinates were set as shown in Fig. 8 of the main text:

$$\mathrm{d}\vec{\mathrm{m}} = \vec{\mathrm{M}}\mathrm{dV}' = \vec{\mathrm{M}}\mathrm{dx}'\mathrm{dy}'\mathrm{dz}' \tag{10}$$

where x', y', z' represent the position of magnetic dipoles and magnetization $\vec{\mathrm{M}}$=(M,0,0).

Therefore, from the basic electromagnetic field law, the magnetic field generated by this infinitely small dipole at point (x, y, z) will be:

$$\mathrm{d}\vec{B}(\vec{r}) = \frac{\mu_0}{4\pi}\left[\frac{3\vec{r}(\vec{M}\cdot\vec{r})}{r^5} - \frac{\vec{M}}{r^3}\right]\mathrm{dx}'\mathrm{dy}'\mathrm{dz}' \tag{11}$$

where $\vec{r} = (x - x', y - y', z - z')$, and μ_0 is the vacuum permeability.

By Integrating over the magnet plates space, each component of the magnetic field at point (x, y, z) can be calculated as:

$$\mathrm{B}_i(x, y, z) = \iiint_{V'} \frac{\mu_0}{4\pi}\left[\frac{3M(x - x')r_i}{r^5} - \frac{M_i}{r^3}\right]\mathrm{dx}'\mathrm{dy}'\mathrm{dz}' \tag{12}$$

where i=x, y, z, and V' are the magnet plates. Therefore, the magnitude and direction of the magnetic field produced by the magnet plates can be determined.

As shown in Fig. 9 in the main text, to calculate the TE properties for the segment L_A of the sample between the magnet plates from the measurement that also included the field-free segment L_B, we assume the two segments L_A and L_B were connected in series. Therefore, the measured total resistivity and Seebeck coefficient, denoted by subscript t, can be expressed as:

$$\begin{gathered}\mathrm{R}_t = 2\mathrm{R}_B + \mathrm{R}_A \\ \mathrm{S}_t\frac{dT}{dL}L_t = 2\mathrm{S}_B\frac{dT}{dL}L_B + \mathrm{S}_A\frac{dT}{dL}L_A\end{gathered} \tag{13}$$

where the symbol R represents electrical resistance Here we assume the thermal gradient was constant through the sample approximately. Therefore, the TE properties between the magnet plates could be calculated as:

$$\begin{gathered}\rho_A = \frac{\rho_t(L_A + 2L_B) - 2L_B\rho_B}{L_A} \\ S_A = \frac{S_t(L_A + 2L_B) - 2L_B S_B}{L_A}\end{gathered} \tag{14}$$

ρ_A and the S_A are the corrected resistivity and Seebeck coefficient shown in Fig. 10 in the main text.

References:

1. Mark Lundstrom. Fundamentals of carrier transport. Cambridge university press, (2009).
2. B. Lenoir, A. Dauscher, M. Cassart, Yu. I. Ravich, and H. Scherrer. *Journal of Physics and Chemistry of Solids*, **59**, 129-134, (1998).
3. Kao Y. H. Cyclotron Resonance Studies of the Fermi Surfaces in Bismuth. *Phys Rev*. **129**(3):1122-1131, (1963).
4. Lenoir B., Scherrer H., Caillat T. Chapter 4, An overview of recent developments for BiSb Alloys. *Semicond Semimetals*. **69**:101-137, (2001).
5. Cutler M., Mott N. F. Observation of Anderson Localization in an Electron Gas. *Phys Rev*. **181**(3):1336-1340, (1969).
